# Predicting Upcoming Events Occurring in the Space Surrounding the Hand

**DOI:** 10.1155/2021/6649135

**Published:** 2021-02-20

**Authors:** Maria L. Rangel, Lidiane Souza, Erika C. Rodrigues, José M. Oliveira, Michelle F. Miranda, Antonio Galves, Claudia D. Vargas

**Affiliations:** ^1^Laboratório de Neurobiologia do Movimento, Instituto Biofísica Carlos Chagas Filho, Universidade Federal do Rio de Janeiro, Rio de Janeiro, Brazil 21941 900; ^2^Núcleo de Pesquisa em Neurociências e Reabilitação, Instituto de Neurologia Deolindo Couto, Universidade Federal do Rio de Janeiro, Rio de Janeiro, Brazil 22290 140; ^3^Universidade Estácio de Sá, Rio de Janeiro, Brazil 26220-099; ^4^Programa de Pós-graduação em Ciências da Reabilitação, Centro Universitário Augusto Motta-UNISUAM, Rio de Janeiro, Brazil 21041 020; ^5^Instituto D'Or de Pesquisa e Ensino IDOR, Rio de Janeiro, Brazil 22281 100; ^6^Instituto de Matemática e Estatística, Universidade de São Paulo, São Paulo, Brazil 05508-090; ^7^Department of Mathematics and Statistics, University of Victoria, Canada

## Abstract

Predicting upcoming sensorimotor events means creating forward estimates of the body and the surrounding world. This ability is a fundamental aspect of skilled motor behavior and requires an accurate and constantly updated representation of the body and the environment. To test whether these prediction mechanisms could be affected by a peripheral injury, we employed an action observation and electroencephalogram (EEG) paradigm to assess the occurrence of prediction markers in anticipation of observed sensorimotor events in healthy and brachial plexus injury (BPI) participants. Nine healthy subjects and six BPI patients watched a series of video clips showing an actor's hand and a colored ball in an egocentric perspective. The color of the ball indicated whether the hand would grasp it (hand movement), or the ball would roll toward the hand and touch it (ball movement), or no event would occur (no movement). In healthy participants, we expected to find distinct electroencephalographic activation patterns (EEG signatures) specific to the prediction of the occurrence of each of these situations. Cluster analysis from EEG signals recorded from electrodes placed over the sensorimotor cortex of control participants showed that predicting either an upcoming hand movement or the occurrence of a tactile event yielded specific neural signatures. In BPI participants, the EEG signals from the sensorimotor cortex contralateral to the dominant hand in the hand movement condition were different compared to the other conditions. Furthermore, there were no differences between ball movement and no movement conditions in the sensorimotor cortex contralateral to the dominant hand, suggesting that BPI blurred specifically the ability to predict upcoming tactile events for the dominant hand. These results highlight the role of the sensorimotor cortex in creating estimates of both actions and tactile interactions in the space around the body and suggest plastic effects on prediction coding following peripheral sensorimotor loss.

## 1. Introduction

Predicting upcoming movements in a variable environment is a fundamental aspect of skilled motor behavior [1–5]. This prediction ability demands an accurate and constantly updated representation of the body and its surrounding space [6, 7] and can be critical for survival [8, 9]. Interestingly, the mere knowledge of a coming action performed by others has been shown to automatically trigger the motor system [8, 10, 11]. Furthermore, the integrity of the parietal cortex has been proven to be important in relation to the capacity to predict upcoming actions [11].

Action observation paradigms have shown that the capacity to estimate the consequences of others' actions seems to be bonded to our own sensorimotor representations [12]. Early seminal work showed the existence of bimodal neurons that were both responsive to tactile stimuli applied to a given body part and to the sight of objects moving towards the same body part in the premotor area F4 of macaque monkeys [13, 14] as well as in the posterior parietal cortex [15, 16]. Such neurons form a network devoted to the representation of peripersonal space, defined as the space directly surrounding different parts of the body [17].

In another series of studies, it was shown that observing other peoples' skin being touched or tickled activated the observer's somatosensory representation in the brain [18–20]. Thus, anticipating the occurrence of a tactile event in the peripersonal body space might trigger specific responses in those brain regions [21, 22]. Furthermore, as suggested by recent behavioral studies, the networks in the brain coding for the space of action (“arm reaching space”) and the peripersonal space could be at least partially segregated. An important finding is that the space within arms' reach is not body part centered, while the peripersonal space is [23]. As a consequence, the predictive coding signatures associated with each of these two networks might also differ.

There is mounting evidence indicating that modifications in the body can alter peripersonal space [24–28]. Among the different types of peripheral injury, brachial plexus injury (BPI) has been seen as a challenging model for the study of brain plasticity [29–35]. Although the upper limb is still connected to the body/trunk, its sensory and motor functions can be deeply impaired due to nerve damage [34]. BPI patients present structural brain change, as well as grey matter atrophy in multiple cortical areas mostly related with motor function [36]. Furthermore, both behavioral [37] and neurophysiological [38] effects have been reported after peripheral lesions affecting the dominant versus nondominant limb. Thus, one could expect that predictive coding aspects that are associated with the peripersonal space might be altered after a BPI in the dominant hand.

The aim of this study was to investigate in healthy participants whether prediction of movement and tactile events occurring in the space surrounding the hand trigger specific electroencephalographic signatures in the sensorimotor cortex. We employed a modified version of the action observation paradigm originally devised by Kilner et al. [10], in which a prediction marker was retrieved from the EEG signals collected over the sensorimotor cortex region when the participants expected to observe a hand moving towards an object. In addition, we introduced a new condition associated specifically with the prediction of an upcoming tactile event, a ball moving towards a hand at rest. We hypothesized that distinct EEG signatures would be associated with the prediction of these upcoming events. If this was the case, then distinct neural networks might be enrolled in coding peripersonal and motor prediction events. Furthermore, in BPI participants, we expected that these EEG signatures would be affected as a function of sensorimotor loss.

## 2. Materials and Methods

### 2.1. Participants

Two groups of right-handed participants were tested: (i) nine neurologically healthy subjects (two women and seven men; mean age 30 years, range 21–49) and (ii) six participants suffering from traumatic unilateral brachial plexus injury (BPI; all males, mean age 28.67 years, range 20–40, see Table 1 for the patients' demographic and clinical data). Handedness was evaluated considering their laterality before the BPI occurrence using the Edinburgh Inventory [39]. All subjects gave informed consent prior to testing. The experiment was approved by the local ethics committee (process number: 298.925, Instituto de Neurologia Deolindo Couto of the Federal University of Rio de Janeiro).

Each patient underwent a neurological evaluation comprising sensory tests using Semmes-Weinstein microfilaments over hand points of exclusive innervation [35]. Muscular manual testing was based on the classification proposed by the Medical Research Council (see Table S1 in the supplementary materials for detailed functional evaluation of BPI participants). The myotomes corresponding to the roots that form the brachial plexus (C5–T1) were assessed [40]. Presence of pain during the experiment was assessed through a numerical verbal scale (0: no pain to 100: acute/sharp pain).

The ability to perform a reaching and grasping movement similar to those observed in the videoclips was tested after the experimental session. All the BPI participants with upper trunk (superior and middle branch) injury were able to reach the ball with their impaired hand, although with compensatory strategies (the quality of movement was not considered, only their ability to perform the movement, see Table S1 in the supplementary material for participant evaluation information). Overall, four participants were diagnosed with a superior trunk BPI on the right side and two participants were diagnosed with complete BPI on the left side (Table 1). One participant reported mild pain during the experimental session.

### 2.2. Experimental Protocol

**Figure 1:**
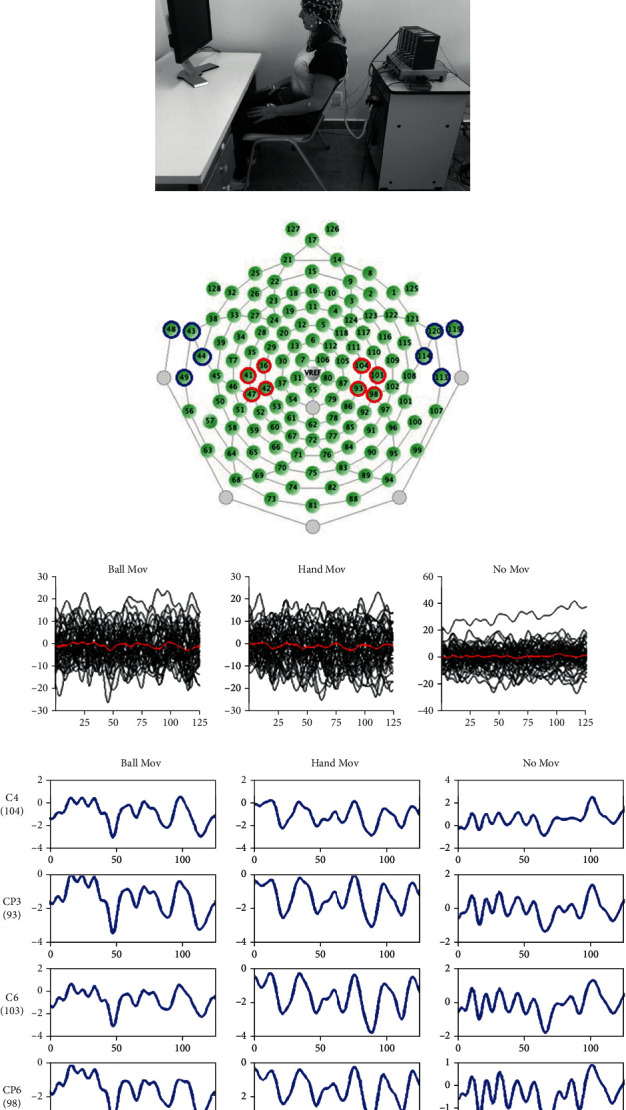
Experimental procedures. (a) The three experimental conditions (no movement (No Mov), ball movement (Ball Mov), and hand movement (Hand Mov)) were presented randomly (total of 60 trials per condition) in a computer screen positioned in front of the participant (b). (c) A dense array geodesic EEG cap depicting in red the target electrodes over the sensorimotor cortex (36, 42, 41, 47, 104, 93, 103, and 98 corresponding to C3, Cp3, C5, Cp5, C4, Cp4, C6, and Cp6, respectively, in 10-10 electrode International System). Control electrodes in the temporal region (48, 43, 44 (corresponding to T9), 49, 113, 114 (corresponding to T10), 119, and 129) are depicted in blue. (d) For each subject and electrode, EEG segments were averaged per condition (signals are represented in millivolts (*y* axis) vs. 125 data points corresponding to 500 ms (*x* axis). (e) These averaged EEG signals were then classified using *k*-means clustering, and Fisher exact-test was performed to test the dependence between condition and cluster label.

The experimental protocol was modified from Kilner et al. [10]. Participants sat comfortably in front of a 17-inch LCD monitor at 60 cm from the screen. EEG signals were recorded while the subjects watched passively a series of video clips presenting an actor's hand and a ball, whose color determined the experimental conditions. Videos were displayed with the software Presentation, version 16.5 (Neurobehavioral Systems, Inc.). All videos started with both the actor's hand and ball halted. In the videos with a yellow ball, 2.5 seconds after the start, the hand moved and grasped the ball (hand movement condition); in the videos with a blue ball, 2.5 seconds after the start, the ball moved toward the actor's hand and touched it (ball movement condition); in videos with a white ball, both the actor's hand and ball remained immobile (no movement condition) (Figure 1 and supplementary 2.). The color rule was explained in advance to the participants by means of verbal instruction. Thus, upcoming events were entirely predictable by the color of the ball at the beginning of each video clip presentation. In order to maintain the participants' attention, one out of eight video clips recorded for each condition contained small changes in hand position and were randomly selected for presentation. In addition, participants were asked to answer a few questions about the videos at the end of the experiment.

Each video clip lasted 3 seconds and was presented 20 times in randomized order, interspersed with a fixation cross on a black screen presented for 1 s (Figure 1(a)). Three blocks of 60 video clips showing the right hand and three blocks of 60 video clips showing the left hand were presented, totaling 60 videos per condition and per hand. Each experimental block lasted about 6 min. There was a rest interval of about 4 min between blocks. The order of presentation of videoclips and blocks was randomized.

During the presentation of the experimental blocks, participants were required to rest their hands in their lap. EMG signals from the first digital interosseous and from the biceps brachialis muscles were monitored bilaterally to detect any hand movement during the task for further trial exclusion upon detection of any hand movement (Figure 1(b)).

### 2.3. Data Acquisition

The EEG signal was acquired with a 128-channel Geodesic Sensor Net coupled with high input impedance amplifier (200 M*Ω*, Net Amps, Electrical Geodesics INC., Eugene, OR, USA), sampled at 500 Hz, and filtered (bandpass filter of 0.3 to 50 Hz). Each electrode impedance was kept below 50 k*Ω*. Electromyographic signals were recorded (MP 100, BIOPAC System) from the first dorsal interosseous (FDI) and biceps brachii (BB) muscles bilaterally, sampled at 1 kHz, amplified (gain: 1000), and filtered (bandpass filter: 10-500 Hz).

### 2.4. Data Analysis

MATLAB 6.5 (Mathworks, USA) was used for the EEG data analysis. The signal was filtered (bandpass filter: 0.3 to 30 Hz) and rereferenced to average auricular electrodes. The signal was presegmented into 60 epochs per experimental condition (hand movement, ball movement, and no movement). A time window of 500 ms (between 2500 and 3000 ms after the start of each video clip) was then selected for analysis. This corresponds to the negative slope time window occurring prior to the movement start [10]. Eye movement and blink artifacts were removed considering a signal amplitude threshold of ±50 mV in the three frontal electrodes (9, 14, and 22 corresponding to Fp2, FpZ, and Fp1 in the 10-10 electrode international positioning system, respectively) and through visual inspection before averaging. EEG was acquired from two sets of electrodes: 8 electrodes in the sensorimotor cortex (36, 42, 41, 47, 104, 93, 103, and 98, corresponding to C3, Cp3, C5, Cp5, C4, Cp4, C6, and Cp6, respectively, using the 10-10 electrode International System, Figure 1(c) in red), and 8 control electrodes in the temporal lobes (48, 43, 44 (corresponding to T9), 49, 113, 114 (corresponding to T10), 119, 129, Figure 1(c) in blue).

### 2.5. Statistical Analysis

The hypothesis that the sensorimotor cortex engages in distinct neural signatures depending on the prediction context was tested. If confirmed, we would be able to distinguish the specific signatures corresponding to each of the three experimental conditions in the EEG segments recorded from the sensorimotor cortex electrodes. To test this hypothesis, we employed a hierarchical approach as follows:

1. For each electrode of interest, we computed the average signal across epochs for each experimental condition (example in Figure 1(d))
2. Next, we considered four sensorimotor cortex electrodes and their corresponding averaged signals from three experimental conditions, and a group of four control electrodes in the temporal cortex and their corresponding averaged signals from three experimental conditions. The electrodes are represented in Figure 1(c), with the control electrodes in blue. For each set of four electrodes and three experimental conditions, the data looks like the example in Figure 1(e)
3. For each subject and each set of electrodes, the 12 averaged signals were submitted to a hierarchical analysis
4. In a first stage, a *k*-means cluster analysis (*k* = 3) for each subject was performed. For each set of electrodes, the goal was to group the 12 averaged signals into three possible clusters: A, B, or C. If the signal in the sensorimotor cortex is different between conditions, the signals from the same condition should belong to the same cluster, with a high separation between the clusters. If the signals in two different conditions belong to the same cluster, there is an indication that this brain region is not recognizing the conditions as distinct from each other
5. In a second stage, the null hypothesis that “the cluster label is independent of the experimental condition” was tested by Fisher's exact test, comparing each pair of conditions for both sets of electrodes. The Fisher exact test is suitable for small sample sizes, and the *p* values of the test can be calculated exactly, rather than relying on an asymptotic approximation of the test statistics. The null hypothesis stated that the cluster label was independent of the experimental condition, while the alternative hypothesis stated that the cluster label was not independent of the experimental condition. Thus, rejection of the null hypothesis meant that it was possible to distinguish between the experimental conditions
6. In the last stage of the hierarchical approach, the Benjamini–Hochberg [41] procedure was used to control the false positive rate in multiple comparisons. Since Fisher's exact test was performed individually within each group of subjects, there is a need to adjust the *p* value accordingly. The adjustment was performed in the statistical software R using the function p.adjust [42].

## 3. Results

The results from the nine controls and the six right-handed BPI participants are presented as contingency tables (Tables 2 and 3). The tables are depicted as a function of the dominance of the hand presented in the videoclip and considering the hemispheres contralateral and ipsilateral to the viewed hand. For most control participants the null hypothesis was rejected for the electrodes over the sensorimotor cortex, indicating that EEG signals coming from the electrodes in the sensorimotor cortex display specific signatures for each of the tested conditions (Table 2). The cluster distinction found between hand movement versus ball movement suggests that at least partially independent neuronal networks code in anticipation of a hand movement versus an object movement directed towards the hand. Finally, no distinction between EEG clusters was found for the electrodes over the temporal cortex.

No cluster difference between the ball movement and no movement conditions was found for any BPI participant in the sensorimotor cortex contralateral to the videos depicting the dominant hand (Table 3). This result indicates that the ability to predict an incoming tactile event in the space surrounding the dominant limb was blurred in these participants.

Cluster analysis further revealed that the contrast between the ball movement and no movement conditions was absent in four of the six tested BPI participants in the hemisphere ipsilateral to the dominant hand. Finally, the difference between ball movement and no movement conditions was preserved in at least four BPI participants, both for the contralateral and the ipsilateral hemispheres in the nondominant hand block. Taken together, these results indicate BPI affected only the ability to estimate upcoming events for the dominant hand.

The comparison between hand movement and no movement conditions when observing the dominant hand showed that the null hypothesis was rejected for the electrodes over the contralateral sensorimotor cortex in four out of six participants. This indicates that the ability to predict an incoming dominant hand movement was preserved in these subjects. However, contrarily to the control subjects, no other difference was found for this comparison condition in the BPI group (Table 3). Likewise, in five of the six BPI participants, the contrast between hand movement and ball movement conditions revealed a difference only for the contralateral sensorimotor cortex during the observation of the dominant hand. The latter result indicates that, in contrast to control participants, the hand movement versus ball movement conditions were indistinguishable in the remaining comparisons (see supplementary tables S6, S7, S8 and S9 for individual results on hypothesis tests). As found in control participants, no distinction between EEG clusters was found for the electrodes over the temporal cortex in BPI participants.

## 4. Discussion

In this study, we investigated the specificity of EEG signatures recorded in anticipation of observing a hand grasping a ball (hand movement), observing a ball touching a hand (ball movement), or observing a stationary hand (no movement) in healthy and in brachial plexus injury (BPI) participants. In healthy participants, the sensorimotor cortex showed a strong dependence between the condition and cluster label irrespective of the observed hand. These results indicate that predicting an upcoming hand movement or predicting the occurrence of a tactile event yields specific neural signatures in the sensorimotor cortex.

In the BPI participants, the hand movement condition differed from the other conditions only for the EEG signals collected in the sensorimotor cortex contralateral to the dominant hand, which, in most cases, was also the affected limb. This result is indicative of a preserved ability to predict others' hand actions. However, no distinction between ball movement and no movement was found in the sensorimotor cortex contralateral to the dominant hand, suggesting that BPI blurred specifically the ability to predict upcoming tactile events for the dominant hand. Interestingly, distinct neural signatures were found for ball movement x no movement conditions for the remaining comparisons, indicating a preserved ability to estimate upcoming tactile events occurring around the nondominant hand.

In contrast with the control group, in the BPI participants, the hand movement condition was not different to the ball movement and no movement conditions for both hemispheres when observing the nondominant hand, as well as for the ipsilateral sensorimotor cortex when observing the dominant hand. Thus, for all these conditions, the neural signatures associated with upcoming hand movements and body envelope events seemed intermingled in the sensorimotor cortex after a BPI.

As expected, both for the control group and the BPI patients, the EEG activity collected in the temporal electrodes did not yield distinct signatures between conditions. Taken together, these results highlight the role of the sensorimotor cortex in creating estimates of both actions and tactile events in the space around the body and suggest plastic effects on this predicting ability following brachial plexus injury.

### 4.1. Estimating Sensorimotor Events

Contemporary neuroscience has consistently shown that predicting other agents' sensorimotor behavior from observation leads to the recruitment of neural circuits similar to those enrolled in their implementation [8]. Kilner et al. [10] investigated whether the readiness potential, traditionally described as an electrophysiological marker of motor preparation, could also be detected whenever an observer expected an upcoming action to occur in a visual display. Results showed the occurrence of a readiness potential when both the nature and onset time of the upcoming action was predictable [10]. Fontana et al. [11] examined whether a readiness potential was generated in chronic stroke patients with focal lesions in the parietal or the premotor cortex when they expected to observe an upcoming movement in a visual display. They found that this prediction marker was preserved in the patients with premotor lesions but abolished in those with parietal lesions, suggesting that the integrity of the parietal cortex is important in relation to the capacity to estimate the occurrence of upcoming actions performed by others.

In the present study, we have shown that the EEG activity associated with the estimation of upcoming hand movements is different from that related to estimating upcoming tactile events in the hand in healthy participants. Both conditions, in turn, were different from the control (no movement) condition. These results indicate that there are specific neural signatures in the sensorimotor cortex for predicting other's touch events. These results are in accordance with the existence of a network devoted specifically to the representation of peripersonal space [13, 16, 17, 43].

The peripersonal space has been proposed to define a safety boundary around the body [44] and a space mediating goal-directed actions [45]. Our results suggest that at least partially separate parietofrontal networks could be at play in anticipating motor or tactile events experienced by others. In a similar manner, employing a set of psychophysical tasks, Zanini et al. [23] argued that the peripersonal space and the within arms' reach space representations are not superimposable. Studies using EEG [46], TMS [47], and fMRI [6, 43, 48] have confirmed that a multisensory representation of peripersonal space takes place in both the parietal and prefrontal areas. As a matter of fact, multisensory integration of both dynamic auditory and looming visual stimuli in the peripersonal space are especially efficient in terms of enhancing an observer's responses to tactile stimulation presented to the body surface [21, 22, 49]. Furthermore, early P100 responses are evoked in the primary somatosensory cortex upon visuotactile synchronic stimulation, irrespectively of whether or not the subject is paying attention to the tactile stimuli, suggesting a preattentive stage of processing [46].

Interestingly, tactile awareness occurs even in the absence of tactile stimulation. Actually, an experience of touch entirely triggered by a visual stimulus can be a relatively common phenomenon, with the predictive processes having a key role in the subjective experience of touch [24]. Since biological systems must face the uncertainty of the environment, the most adaptive responses are those which succeed in minimizing the cost of the surprise effect. The best way to achieve this is to develop a system capable of anticipating, through preattentive processes, the most probable events in a certain context [1, 35, 50, 51]. The neural signatures described herein for upcoming tactile events in others' hands may be a physiological correlate of a predictive mechanism specifically devoted to body envelope events.

### 4.2. Plastic Changes after BPI Affects Predictive Coding

It has been widely demonstrated that lesions in the body are capable of promoting structural and functional modifications in the sensory (S1) and motor (M1) primary cortices [52]. Likewise, long-lasting changes in the body envelope caused by traumatic BPI should be expected to lead to modifications in the brain. Mano et al. [53] and Malessy et al. [31] were pioneers in investigating cortical plasticity in BPI. Later on, employing resting-state fMRI, Fraiman et al. [33] found evidence that these modifications encompass the M1 trunk/lower limb representation, suggesting that BPI might imply a bodily extended motor dysfunction. Accordingly, it was found that BPI affects body balance [54]. Ramalho et al. [35] showed that a unilateral BPI impairs bilateral touch threshold, suggesting that higher order mechanisms of plasticity are at play after a BPI. Moreover, plasticity after BPI does not seem to be restricted to the sensorimotor cortex, involving regions such as the precuneus, the lateral aspect of the posterior parietal cortex, the superior parietal lobe, and the intraparietal sulcus [37]. Thus, we expected that traumatic BPI would also lead to changes in the brain signatures associated with the detection of upcoming motor and tactile events.

In a small sample of six BPI participants, we found evidence of preserved capacity to predict upcoming movements of the dominant hand. Four of them had an upper trunk BPI in the dominant hand. The remaining two participants had complete BPI in the nondominant hand. Rodrigues et al. (2008) reported the loss of a prediction marker associated with upcoming movements in the hemisphere contralateral to the injured limb in unilateral amputees [55]. Thus, a parsimonious explanation could be that the predictive coding associated specifically with the goal of the task (grasping the ball) was preserved in BPI patients as a consequence of a “motorically” preserved hand, even though the arm was rendered immobile by the BPI. There were no further differences between hand movement, ball movement, or no movement conditions in any of the remaining comparisons and for none of the tested BPI participants. Although limited by the reduced number of BPI participants, these results suggest that the plastic reorganization after BPI is associated with modifications in motor planning at a higher level. As a matter of fact, a resting-state FMRi connectivity study showed that the dominant hand disability seems to trigger changes not only in sensorimotor but also in higher order areas [56]. In BPI participants, the default mode and executive control networks functional connectivity were abnormally synchronized, possibly resulting in inefficient performance. Thus, a cascade functional remodeling might have occurred after severe peripheral nerve injury [56].

Compelling evidence points to plastic changes in both hemispheres after a unilateral BPI [33, 35, 57]. Interestingly, a study found that slower reaction times and reduced accuracy in mental imagery tasks are associated with amputation of the dominant limb compared to amputation of the nondominant limb. The authors suggested that the loss of a dominant upper limb might degrade the efficiency of both dominant and nondominant limb motor behaviors and imagery at the motor preparation level [37]. Event-related potentials measured in long-standing right hand amputees while they performed a mental rotation task revealed a bilateral decrease in N200 during the categorization phase whose magnitude was correlated with the time of amputation [58]. Furthermore, amputees displayed an increase in P300 in the hemisphere contralateral to the intact limb during the mental rotation phase that was interpreted as resulting from a change in hand dominance [58]. In our sample, BPI had occurred relatively recently at the time of testing (up to two years). Patients with right side injury were still able to use their dominant/right hand, which may have contributed to the maintenance of a vivid motoric representation of the hand in the left (dominant) sensorimotor cortex. Accordingly, our results show that four of the six tested BPI participants did present a neural signature associated with predictive coding of movements of the dominant hand. Further studies are necessary to understand the role of hand dominance in the predictive coding of hand movements after a peripheral lesion.

BPI also specifically blurred the prediction marker associated with an incoming tactile event in the hemisphere contralateral to the dominant hand. The difference between ball movement and no movement conditions was, however, preserved for the remaining comparisons. Traumatic BPI often leads to severe impairment of tactile threshold detection throughout the affected limb [35]. Moreover, the peripersonal space representation was shown to be body centered [6, 17, 22], continuously recalibrating upon receiving updated environmental information [59]. By impairing this sensory updating, BPI would blur the predictive mechanisms relating to upcoming tactile events in the space surrounding the hand.

The shape and size of peripersonal space are not fixed, but instead adapt as a function of interaction with the environment. In a seminal work, Iriki et al. [15] have shown in monkeys that the active reach for a piece of food with a tool expands the visual receptive field of neurons in the intraparietal sulcus. In humans, the peripersonal space extends in space after using a tool to reach far locations [60]. Previous research has shown that voluntary object-oriented actions induce an online, continuous remapping of the peripersonal space of the hand, evidence that supports a role for this space in the guidance of actions [43, 61]. On the other hand, the mere immobilization of the upper limb has been shown to shrink the boundaries of peripersonal space, suggesting a fundamental role of physical constraint over space representation [25]. Thus, peripersonal space representations seem to be highly dependent on ongoing behavior.

In BPI, the physical presence of the injured limb attached to the body may play an important role in modifying peripersonal space representation. Indeed, it has been shown that traumatic upper limb amputation leads to an asymmetry in the upper limb space of action, expressed by a distortion in the visuospatial perception of the affected limb [27]. Interestingly, the implicit perception of limb size and the peripersonal space representation surrounding the amputated limb are restored by the use of a prosthesis [62]. Further research might shed light on higher order peripersonal modifications induced by peripheral lesions.

## 5. Conclusions

Cluster analysis from EEG signals recorded from the sensorimotor cortex of control participants indicates that specific neural signatures are associated with the prediction of an upcoming hand action or the occurrence of a tactile event. For BPI participants, predicting actions for the dominant hand yielded distinct activity in the contralateral sensorimotor cortex, an indication of preserved ability to predict others' hand actions. Conversely, the ability to code for an upcoming tactile event was abolished for this hand, suggesting a dependency of the online sensory information to estimate events in the hand space. Despite the small sample size, our results draw attention to the role of the sensorimotor cortex in creating estimates of both actions and tactile interactions in the space around the body and suggest plastic effects on predicting ability following peripheral sensorimotor loss.

## Figures and Tables

**Table 1:** Characteristics of participants with BPI.

ID	Age	Handedness	Injury side	Lesion	Time since injury (months)
BPI1	30	R	R	S, M	15
BPI2	20	R	R	S, M	8
BPI3	24	R	R	S, M	7
BPI4	32	R	R	S, M	8
BPI5	26	R	L	S, M, I	24
BPI6	40	R	L	S, M, I	6

Anatomical localization of BPI: S: superior trunk; M: middle trunk; I: inferior trunk; R: right; L: left.

**Table 2:** Contingency table showing the number of rejections of H0 for control participants' hypotheses tests (see also Tables S2, S3, S4 and S5 for individual results).

Condition comparison	Control (*n* = 9)			
	Dominant hand view		Nondominant hand view	
	C. H.	I. H.	C. H.	I. H.
Ball Mov x No Mov	7	9	9	9
Hand Mov x No Mov	8	8	9	8
Hand Mov x Ball Mov	8	7	9	6

C.H.: hemisphere contralateral to the observed hand; I.H.: hemisphere ipsilateral to the observed hand.

**Table 3:** Contingency table showing the number of rejections of H0 for BPI participants' hypotheses tests (see also Tables S6, S7, S8 and S9 for individual results).

*Condition* *Comparison*	BPI patients (n =6)			
	Dominant^∗^ hand view		Nondominant^∗^ hand view	
	C. H.	I. H.	C. H.	I. H.
Ball Mov x No Mov	0	4	4	5
Hand Mov x No Mov	4	0	0	0
Hand Mov x Ball Mov	5	0	0	0

C.H.: hemisphere contralateral to the observed hand; I.H.: hemisphere ipsilateral to the observed hand. ^∗^All the participants were right-handed before the BPI injury.

## Data Availability

The EEG data used to support the findings of this study are available on request. Please contact the corresponding author for more information.

## Supplementary Materials

Supplementary 1 Table S1: BPI functional evaluation of BPI participants. Table S2: contingency table showing the *p* values of Fisher's exact tests for control participants in the dominant hand observation. Table S3: contingency table showing the adjusted *p* values of the Fisher's exact tests for control participants in the dominant hand observation. Table S4: Contingency table showing the *p* values of Fisher's exact tests for each control participant in the nondominant hand observation. Table S5: contingency table showing the adjusted *p* values of Fisher's exact tests for each control participant in the nondominant hand observation. Table S6: contingency table showing the *p* values of Fisher's exact tests for each BPI participant in the dominant hand observation. Table S7: contingency table showing the adjusted *p* values of Fisher's exact tests for each BPI participant in the dominant hand observation. Table S8: contingency table showing the *p* values of Fisher's exact tests for each BPI patient in the nondominant hand observation. Table S9: contingency table showing the adjusted *p* values of Fisher's exact tests for each BPI patient in the nondominant hand observation.

Supplementary 2 Supplementary video 1 Hand movement, ball movement, and no movement conditions employed in the experiment: https://drive.google.com/file/d/1IZgeVNrckUPtCAUS5ks8QqMwRvZtJHLG/view?usp=drivesdk.
