# Supplementary material for: Predicting Upcoming Events Occurring in the Space Surrounding the Hand
Source: Neural Plast. 2021 Feb 20;2021:6649135. doi: 10.1155/2021/6649135 (PMC7914383; doi:10.1155/2021/6649135)
Supplement: Supplementary 1 — Table S1: BPI functional evaluation of BPI participants. Table S2: contingency table showing the p values of Fisher's exact tests for control participants in the dominant hand observation. Table S3: contingency table showing the adjusted p values of the Fisher's exact tests for control participants in the dominant hand observation. Table S4: Contingency table showing the p values of Fisher's exact tests for each control participant in the nondominant hand observation. Table S5: contingency table showing the adjusted p values of Fisher's exact tests for each control participant in the nondominant hand observation. Table S6: contingency table showing the p values of Fisher's exact tests for each BPI participant in the dominant hand observation. Table S7: contingency table showing the adjusted p values of Fisher's exact tests for each BPI participant in the dominant hand observation. Table S8: contingency table showing the p values of Fisher's exact tests for each BPI patient in the nondominant hand observation. Table S9: contingency table showing the adjusted p values of Fisher's exact tests for each BPI patient in the nondominant hand observation. [file 6649135.f1.docx]

Table S1 – Functional evaluation of BPI participants.

|  | *Muscular Manual Test* | | | | | *Sensory Test* | | | | | *R&G*  *ability* | *Pain*  *NVS* |
| --- | --- | --- | --- | --- | --- | --- | --- | --- | --- | --- | --- | --- |
|  | C5 | C6 | C7 | C8 | T1 | Median | | Radial | Ulnar | |  |  |
| BPI01 | 1 | 3 | 0 | 5 | 3 | 3.78 | 5.57 | | 4.11 | Yes | | 0 |
| BPI02 | 3 | 5 | 4 | 5 | 5 | NT | NT | | NT | Yes | | 0 |
| BPI03 | 1 | 4 | 4 | 5 | 4 | 3.6 | 4.65 | | 2.6 | Yes | | 0 |
| BPI04 | 1 | 4 | 5 | 4 | 4 | 5.26 | 4.65 | | 3.6 | Yes | | 0 |
| BPI05 | 2 | 0 | 1 | 0 | 0 | A* | A* | | A* | No | | 0 |
| BPI06 | 0 | 0 | 0 | 0 | 0 | A* | A* | | 5.57 | No | | 50 |

Table S1 - Muscular Manual Test (0- No visible or palpable contraction; 1 - Visible or palpable contraction with no motion; 2 - Full range of movement when positioned with gravity eliminated; 3 – Full range of movement against gravity; 4 - Full range of movement against gravity, moderate resistance; 5 - Full ROM against gravity, maximum resistance). Sensory test with Semmes Weinstein Monofilaments ranging from 1.84 to 6.20, as expressed in log 10 × F; with F = force in milligram. NT – not tested; A* - Absent (see Ramalho et al., 2019 for details). R&G ability - Reaching and Grasping ability - Qualitative analyses for reaching and grasping with injured limb (Yes – able to reach and grasp a ball; No – not able to reach and grasp a ball); Pain Numerical Verbal Scale (NVS) (0:No pain --100: worst pain)

Table S2 - Contingency table showing the p-values of the Fisher's exact tests for control participants in the dominant hand observation

| Participant ID | Ball Mov x No Mov | | Hand Mov x No Mov | | Ball Mov x Hand Mov | |
| --- | --- | --- | --- | --- | --- | --- |
|  | Contralateral Hemisphere | Ipsilateral Hemisphere | Contralateral Hemisphere | Ipsilateral Hemisphere | Contralateral Hemisphere | Ipsilateral Hemisphere |
| S01 | **0.03180** | **0.03070** | **0.02770** | **0.02670** | **0.02640** | **0.02930** |
| S02 | **0.02620** | **0.02870** | 0.14369 | **0.02610** | **0.02450** | **0.02980** |
| S03 | **0.02690** | **0.02730** | **0.02700** | **0.02790** | **0.02560** | **0.02970** |
| S04 | 0.43566 | **0.02980** | **0.02980** | 1.00000 | **0.02760** | **0.02840** |
| S05 | **0.03120** | **0.02890** | **0.02770** | **0.03060** | **0.02970** | **0.03170** |
| S06 | 0.14079 | **0.02750** | **0.02900** | **0.03090** | **0.02870** | 1.00000 |
| S07 | **0.02750** | **0.02620** | **0.02710** | **0.03190** | 0.14309 | 0.13839 |
| S08 | **0.02850** | **0.02960** | **0.02730** | **0.02790** | **0.03040** | **0.02650** |
| S09 | **0.02660** | **0.02820** | **0.02950** | **0.03170** | **0.02890** | **0.03110** |
| H0 Rejections | 7/9 | 9/9 | 8/9 | 8/9 | 9/9 | 7/9 |

Table S2 - Comparisons between condition pairs showing the p-values of the Fisher's exact tests. Bold indicates the rejection of the null hypothesis (H0). For this participant and hemisphere it was possible to distinguish between the two experimental conditions. *BPI05 and BPI06 had a complete BPI in the left (non-dominant) limb.

Table S3 - Contingency table showing the adjusted p-values of the Fisher's exact tests for control participants in the dominant hand observation

| Participant ID | Ball Mov x No Mov | | Hand Mov x No Mov | | Ball Mov x Hand Mov | |
| --- | --- | --- | --- | --- | --- | --- |
|  | Contralateral Hemisphere | Ipsilateral Hemisphere | Contralateral Hemisphere | Ipsilateral Hemisphere | Contralateral Hemisphere | Ipsilateral Hemisphere |
| S01 | **0.04089** | **0.03070** | **0.03353** | **0.03589** | **0.03420** | **0.04076** |
| S02 | **0.04089** | **0.03070** | 0.14369 | **0.03589** | **0.03420** | **0.04076** |
| S03 | **0.04089** | **0.03070** | **0.03353** | **0.03589** | **0.03420** | **0.04076** |
| S04 | 0.43566 | **0.03070** | **0.03353** | 1.00000 | **0.03420** | **0.04076** |
| S05 | **0.04089** | **0.03070** | **0.03353** | **0.03589** | **0.03420** | **0.04076** |
| S06 | 0.15839 | **0.03070** | **0.03353** | **0.03589** | **0.03420** | 1.00000 |
| S07 | **0.04089** | **0.03070** | **0.03353** | **0.03589** | 0.14309 | 0.15569 |
| S08 | **0.04089** | **0.03070** | **0.03353** | **0.03589** | **0.03420** | **0.04076** |
| S09 | **0.04089** | **0.03070** | **0.03353** | **0.03589** | **0.03420** | **0.04076** |
| H0 Rejections | 7/9 | 9/9 | 8/9 | 8/9 | 9/9 | 7/9 |

Table S3 - The Benjamini–Hochberg procedure was used to control the false positive rate in multiple comparisons. Since the Fisher’s exact test was performed individually within each group of subjects, there is a need to adjust the p-value accordingly. The adjustment was performed in the statistical software R using the function p.adjust. Bold indicates the rejection of the null hypothesis (H0). For this participant and hemisphere it was possible to distinguish between the two experimental conditions.

Table S4 - Contingency table showing the p-values of the Fisher's exact tests for control participants in the non-dominant hand observation

| Participant ID | Ball Mov x No Mov | | Hand Mov x No Mov | | Ball Mov x Hand Mov | |
| --- | --- | --- | --- | --- | --- | --- |
|  | Contralateral Hemisphere | Ipsilateral Hemisphere | Contralateral Hemisphere | Ipsilateral Hemisphere | Contralateral Hemisphere | Ipsilateral Hemisphere |
| S01 | **0.02870** | **0.03270** | 0.14529 | **0.02780** | **0.02800** | **0.02790** |
| S02 | **0.02780** | **0.02690** | **0.02920** | **0.02860** | **0.03150** | **0.03000** |
| S03 | **0.02590** | **0.02820** | **0.02620** | **0.02800** | **0.03270** | **0.03020** |
| S04 | **0.02850** | **0.02560** | **0.03130** | 0.23438 | **0.02760** | 1.00000 |
| S05 | **0.02750** | **0.02810** | **0.02930** | **0.02850** | **0.02880** | **0.02810** |
| S06 | **0.02880** | **0.03130** | **0.02940** | **0.02710** | **0.02780** | **0.02860** |
| S07 | **0.02740** | **0.03220** | **0.02970** | **0.03070** | **0.02900** | 0.14179 |
| S08 | **0.02920** | **0.02860** | **0.02800** | **0.02760** | **0.02820** | **0.02680** |
| S09 | **0.02890** | **0.02880** | **0.02910** | **0.02870** | **0.02760** | 0.13869 |
| H0 Rejections | 9/9 | 9/9 | 8/9 | 8/9 | 9/9 | 6/9 |

Table S4 - Comparisons between condition pairs showing the p-values of the Fisher's exact tests. Bold indicates the rejection of the null hypothesis (H0). For this participant and hemisphere it was possible to distinguish between the two experimental conditions.

Table S5 - Contingency table showing the adjusted p-values of the Fisher's exact tests for control participants in the non-dominant hand observation

| Participant ID | Ball Mov x No Mov | | Hand Mov x No Mov | | Ball Mov x Hand Mov | |
| --- | --- | --- | --- | --- | --- | --- |
|  | Contralateral Hemisphere | Ipsilateral Hemisphere | Contralateral Hemisphere | Ipsilateral Hemisphere | Contralateral Hemisphere | Ipsilateral Hemisphere |
| S01 | **0.02920** | **0.03270** | 0.14529 | **0.03454** | **0.03270** | **0.04530** |
| S02 | **0.02920** | **0.03270** | **0.03521** | **0.03454** | **0.03270** | **0.04530** |
| S03 | **0.02920** | **0.03270** | **0.03521** | **0.03454** | **0.03270** | **0.04530** |
| S04 | **0.02920** | **0.03270** | **0.03521** | 0.23438 | **0.03270** | 1.00000 |
| S05 | **0.02920** | **0.03270** | **0.03521** | **0.03454** | **0.03270** | **0.04530** |
| S06 | **0.02920** | **0.03270** | **0.03521** | **0.03454** | **0.03270** | **0.04530** |
| S07 | **0.02920** | **0.03270** | **0.03521** | **0.03454** | **0.03270** | 0.15951 |
| S08 | **0.02920** | **0.03270** | **0.03521** | **0.03454** | **0.03270** | **0.04530** |
| S09 | **0.02920** | **0.03270** | **0.03521** | **0.03454** | **0.03270** | 0.15951 |
| H0 Rejections | 9/9 | 9/9 | 8/9 | 8/9 | 9/9 | 6/9 |

Table S5 - The Benjamini–Hochberg procedure was used to control the false positive rate in multiple comparisons. Since the Fisher’sexact test was performed individually within each group of subjects, there is a need to adjust the p-value accordingly. The adjustment was performed in the statistical software R using the function p.adjust. Bold indicates the rejection of the null hypothesis (H0). For this participant and hemisphere it was possible to distinguish between the two experimental conditions.

Table S6 Contingency table showing the p-values of the Fisher's exact tests for BPI participants in the dominant hand observation.

| Participant ID | Ball Mov x No Mov | | Hand Mov x No Mov | | Ball Mov x Hand Mov | |
| --- | --- | --- | --- | --- | --- | --- |
|  | Contralateral Hemisphere | Ipsilateral Hemisphere | Contralateral Hemisphere | Ipsilateral Hemisphere | Contralateral Hemisphere | Ipsilateral Hemisphere |
| BPI01 | 0.02830 | **0.02949** | **0.02610** | 0.02960 | 0.14279 | 0.03000 |
| BPI02 | 0.02860 | **0.02890** | **0.02900** | 0.02430 | **0.02730** | 0.02610 |
| BPI03 | 0.14069 | **0.02390** | **0.02580** | 0.14199 | **0.02490** | 0.03100 |
| BPI04 | 1.00000 | 0.13839 | 0.23098 | 0.13879 | **0.03050** | 0.65893 |
| BPI05* | 0.02780 | **0.02810** | **0.02840** | 0.02580 | **0.03000** | 0.48515 |
| BPI06* | 0.14179 | 1.00000 | 0.13809 | 0.14369 | **0.02940** | 0.14989 |
| H0 Rejections | 0/6 | 4/6 | 4/6 | 0/6 | 5/6 | 0/6 |

Table S6 - Comparisons between condition pairs showing the p-values of the Fisher's exact tests. Bold indicates the rejection of the null hypothesis (H0). For this participant and hemisphere it was possible to distinguish between the two experimental conditions. *BPI05 and BPI06 had a complete BPI in the left (non-dominant) limb.

Table S7 Contingency table showing the adjusted p-values of the Fisher's exact tests for BPI participants in the dominant hand observation.

| Participant ID | Ball Mov x No Mov | | Hand Mov x No Mov | | Ball Mov x Hand Mov | |
| --- | --- | --- | --- | --- | --- | --- |
|  | Contralateral Hemisphere | Ipsilateral Hemisphere | Contralateral Hemisphere | Ipsilateral Hemisphere | Contralateral Hemisphere | Ipsilateral Hemisphere |
| BPI01 | 0.05720 | **0.04425** | **0.04350** | 0.05920 | 0.14279 | 0.06200 |
| BPI02 | 0.05720 | **0.04425** | **0.04350** | 0.05920 | **0.03660** | 0.06200 |
| BPI03 | 0.17015 | **0.04425** | **0.04350** | 0.14369 | **0.03660** | 0.06200 |
| BPI04 | 1.00000 | 0.16607 | 0.23098 | 0.14369 | **0.03660** | 0.65893 |
| BPI05* | 0.05720 | **0.04425** | **0.04350** | 0.05920 | **0.03660** | 0.58218 |
| BPI06* | 0.17015 | 1.00000 | 0.16571 | 0.14369 | **0.03660** | 0.22484 |
| H0 Rejections | 0/6 | 4/6 | 4/6 | 0/6 | 5/6 | 0/6 |

Table S7 - The Benjamini–Hochberg precodure was used to control the false positive rate in multiple comparisons. Since the Fisher’s exact test was performed individually within each group of subjects, there is a need to adjust the p-value accordingly. The adjustment was performed in the statistical software R using the function p.adjust. Bold indicates the rejection of the null hypothesis (H0). For this participant and hemisphere it was possible to distinguish between the two experimental conditions. *BPI05 and BPI06 had a complete BPI in the left (non-dominant) limb

Table S8 – Contingency table showing the p-values of the Fisher's exact tests for BPI participants in the non-dominant hand observation.

| Participant ID | Ball Mov xv No Mo | | Hand Mov x No mov | | Hand Mov x Ball Mov | | |
| --- | --- | --- | --- | --- | --- | --- | --- |
|  | Contralateral Hemisphere | Ipsilateral Hemisphere | Contralateral Hemisphere | Ipsilateral Hemisphere | Contralateral Hemisphere | | Ipsilateral Hemisphere |
| BPI01 | **0.02850** | **0.02790** | 0.02890 | 0.03180 | 1.00000 | 0.02920 | |
| BPI02 | **0.02980** | **0.02790** | 0.03520 | 0.02580 | 0.02670 | 0.03050 | |
| BPI03 | 0.48815 | 0.14769 | 0.65943 | 0.05639 | 0.14419 | 0.48585 | |
| BPI04 | **0.03010** | **0.02840** | 0.05869 | 0.13889 | 1.00000 | 0.43536 | |
| BPI05* | **0.02500** | **0.02920** | 0.02760 | 0.02850 | 1.00000 | 0.02860 | |
| BPI06* | 1.00000 | **0.02900** | 0.02900 | 1.00000 | 0.22568 | 0.05709 | |
| H0 Rejections | 4/6 | 5/6 | 0/6 | 0/6 | 0/6 | 0/6 | |

Table S8 - Comparisons between condition pairs showing the p-values of the Fisher's exact tests. Bold indicates the rejection of the null hypothesis (H0). For this participant and hemisphere it was possible to distinguish between the two experimental conditions. *BPI05 and BPI06 had a complete BPI in the left (non-dominant) limb.

Table S9 - Contingency table showing the adjusted p-values of the Fisher's exact tests for BPI participants in the non-dominant hand observation.

| Participant ID | Ball Mov x No Mov | | Hand Mov x No mov | | Hand Mov x Ball Mov | | |
| --- | --- | --- | --- | --- | --- | --- | --- |
|  | Contralateral Hemisphere | Ipsilateral Hemisphere | Contralateral Hemisphere | Ipsilateral Hemisphere | Contralateral Hemisphere | | Ipsilateral Hemisphere |
| BPI01 | **0.04515** | **0.03504** | 0.05280 | 0.06360 | 1.00000 | 0.06100 | |
| BPI02 | **0.04515** | **0.03504** | 0.05280 | 0.06360 | 0.16020 | 0.06100 | |
| BPI03 | 0.58578 | 0.14769 | 0.65943 | 0.08459 | 0.43257 | 0.48585 | |
| BPI04 | **0.04515** | **0.03504** | 0.07043 | 0.16667 | 1.00000 | 0.48585 | |
| BPI05* | **0.04515** | **0.03504** | 0.05280 | 0.06360 | 1.00000 | 0.06100 | |
| BPI06* | 1.00000 | **0.03504** | 0.05280 | 1.00000 | 0.45136 | 0.08564 | |
| H0 Rejections | 4/6 | 5/6 | 0/6 | 0/6 | 0/6 | 0/6 | |

Table S9 - The Benjamini–Hochberg procedure was used to control the false positive rate in multiple comparisons. Since the Fischer’s exact test was performed individually within each group of subjects, there is a need to adjust the p-value accordingly. The adjustment was performed in the statistical software R using the function p.adjust. Bold indicates the rejection of the null hypothesis (H0). For this participant and hemisphere it was possible to distinguish between the two experimental conditions. *BPI05 and BPI06 had a complete BPI in the left (non-dominant) limb.
